# JSI-124 Suppresses Invasion and Angiogenesis of Glioblastoma Cells *In Vitro*

**DOI:** 10.1371/journal.pone.0118894

**Published:** 2015-03-19

**Authors:** Guang Yuan, Shaofeng Yan, Hao Xue, Ping Zhang, Jintang Sun, Gang Li

**Affiliations:** 1 Department of Neurosurgery, Qilu Hospital of Shandong University, 107 Wenhua Xi Road, Jinan, 250012, P.R. China; 2 Brain Science Research Institute, Shandong University, 44 Wenhua Xi Road, Jinan, 250012, P.R. China; 3 Institute of Basic Medical Sciences and Key Laboratory of Cardiovascular Proteomics of Shandong Province, Qilu Hospital of Shandong University, 44 Wenhua Xi Road, Jinan, 250012, P.R. China; 4 Department of Neurosurgery, Central Hospital of Zibo City, 54 Gongqingtuan Xi Road, Zibo, 255036, P.R. China; Medical College of Wisconsin, UNITED STATES

## Abstract

Glioblastoma multiforme (GBM) is one of the utmost malignant tumors. Excessive angiogenesis and invasiveness are the major reasons for their uncontrolled growth and resistance toward conventional strategies resulting in poor prognosis. In this study, we found that low-dose JSI-124 reduced invasiveness and tumorigenicity of GBM cells. JSI-124 effectively inhibited VEGF expression in GBM cells. In a coculture study, JSI-124 completely prevented U87MG cell–mediated capillary formation of HUVECs and the migration of HUVECs when cultured alone or cocultured with U87MG cells. Furthermore, JSI-124 inhibited VEGF-induced cell proliferation, motility, invasion and the formation of capillary-like structures in HUVECs in a dose-dependent manner. JSI-124 suppressed VEGF-induced p-VEGFR2 activity through STAT3 signaling cascade in HUVECs. Immunohistochemistry analysis showed that the expression of CD34, Ki67, p-STAT3 and p-VEGFR2 protein in xenografts was remarkably decreased. Taken together, our findings provide the first evidence that JSI-124 effectively inhibits tumor angiogenesis and invasion, which might be a viable drug in anti-angiogenesis and anti-invasion therapies.

## Introduction

Glioblastoma multiforme (GBM), the most aggressive and accounts for 54% of all gliomas [1], is considered incurable largely due to sustained and excessive angiogenesis and invasiveness, and approximately 77% of glioma patients die within the first year of their diagnosis [2–4]. Angiogenesis, considered crucial for the transition of tumors from a dormant to malignant state [5,6], is now established as one of the hallmarks of cancer and responsible for over 90% of all cancer deaths [7]. Angiogenesis is a rate-limiting process including the destabilization of integrated blood vessel, endothelial cell proliferation, migration, and tubulogenesis. Disrupting tumor angiogenesis has been shown effective tumor growth and metastasis inhibition [8].

Moreover, accumulating evidence shows that the STAT3 is highly expressed in manlignant gliomas and strongly linked to tumor angiogenesis and metastasis [9–12]. As a latent self-signaling transcription factor, STAT3 is activated by certain interleukins and growth factors. Compelling evidence has established that constitutive and aberrant activation of STAT3 occur in malignant gliomas and play a pivotal role in malignant transformation, tumor cell survival and angiogenesis [13]. Furthermore, recent studies have identified STAT3 as a direct transcriptional activator of VEGF and hypoxia- inducible factor 1α (HIF-1α) under hypoxia, which are key stimuli known to initiate endothelial cell migration, invasion and differentiation [14]. Activated STAT3 leads to transcription of various target genes, such as cyclin D1, Bcl-2, Bcl-xL, matrix metalloproteinase 2 (MMP2), and VEGF, to regulate cell survival, angiogenesis, immune evasion, and inflammation in tumor microenvironment [15,16]. Inhibiting activated STAT3 signaling contributes to angiogenesis inhibition, tumor growth arrest, and metastasis suppression [17–19]. Currently, several strategies have been already reported to block the action of STAT3 pathway, including natural compounds, peptidomimetic compounds, small molecules, and oligonucleotides which have been developed and are undergoing into clinical stages [8,20]. Therefore, agents that interfere with activated STAT3 are promising for prevention and treatment of cancer.

JSI-124 (cucurbitacin I), a natural chemical compound belonging to the cucurbitacin family, was discovered as a potent STAT3 inhibitor and exhibited anticancer potential through the induction of apoptosis in a wide variety of human tumor cell lines in multiple cancer cell lines, such as breast cancer, lung cancer, glioma, and melanoma [19,21,22]. However, the exact mechanism of JSI-124 is not fully elucidated.

In this study, we screened a number of natural compounds and found that JSI-124 exerted its invasion inhibition property at low dose and its anti-angiogenesis characteristic. We provide evidence that JSI-124 dose dependently suppresses the activation of STAT3 in human endothelial cells. Our results indicate that JSI-124 could potentially be beneficial as a promising therapeutic agent for GBM.

## Materials and Methods

### Ethics Statement

The experiments conformed to the Animal Management Rule of the Chinese Ministry of Health (documentation 55, 2001), and the experimental protocol was approved by the Animal Care and Use Committee of Shandong University.

### Reagents

JSI-124 (Cucurbitacin I) was purchased from Sigma. A 1 mg/ml JSI-124 stock solution was prepared in dimethyl sulfoxide (DMSO; Sigma), stored at −20°C and then diluted as needed in cell culture medium. Recombinant human VEGF_165_ was purchased from R&D Systems. Matrigel and transwell chambers were obtained from BD Biosciences. Antibodies against JAK2, STAT3, phospho-STAT3 (Ser727),VEGFR2, phospho-VEGFR2 (Tyr1175), Bcl-2, Bcl-xL, Caspase-3, GAPDH and poly (ADP-ribose) polymerase (PARP) were obtained from Cell Signaling Technology. Phospho-JAK2 (Y1007/Y1008) was purchased from Abcam.

### Cell lines and cell culture

Human umbilical vein endothelial cells (HUVECs) were obtained from the American Type Culture Collection (ATCC). HUVECs were cultured in endothelial cell medium (ECM):M199 medium (Life Technologies, Invitrogen) supplemented with 20% fetal bovine serum (Hyclone, USA), 20 μg/mL bovine endothelial cell growth factor (Roche), 0.1 mg/mL heparin (Sigma) at 37°C with 5% CO2. All human glioblastoma cells were obtained from ATCC and incubated in DMEM (GIBCO, USA) supplemented with 10% fetal bovine serum (Hyclone, USA), 100 units/ml penicillin, and 100μg/ml streptomycin in a humidified air of 5% CO2 at 37°C.

### Cell viability assay

The cytotoxic effect of JSI-124 on GBM cells and HUVECs were determined using CCK-8 assay (Dojindo, Japan). Cells in medium containing 20% FBS or 10% FBS were seeded into 96-well flat-bottomed plates at 5×10^3^ cells/well and incubated at 37°C overnight. After the desired treatment, the cells were incubated for an additional 4 h with 100μl serum free DMEM with 10μl CCK-8 at 37°C. The absorbance at 450 nm was measured using a microplate reader. The absorbance was measured at 450 nm wavelength.

### Western blot analysis

After the desired treatment, cells were washed twice with cold phosphate-buffered saline (PBS) and harvested with a rubber scraper. Cell pellets were lysed and kept on ice for at least 30 min in a buffer containing 50 mM TrisHCl (pH 7.4), 150 mM NaCl, 0.5% Nonidet P-40, 50 mM NaF, 1 mM Na_3_VO_4_, 1 mM phenylmethylsulfonyl fluoride and 1mM PMSF. The lysates were cleared by centrifugation and the supernatants were collected. Cell lysates were then separated by sodium dodecyl sulfate-polyacrylamide gel electrophoresis (SDS-PAGE) and subjected to western blot analysis with the primary antibodies and horseradish peroxidase-labeled secondary antibodies.

### VEGF Enzyme-Linked Immunosorbent Assay

The VEGF protein that released into the conditioned medium of U87MG cells was determined using an ELISA kit (R&D Systems, Minneapolis, MN, USA). U87MG cells (5×10^5^) were seeded in six-well plates in 2ml of complete growth medium. Twenty-four hours later, cells were serum-starved for 24 hours and then exposed to JSI-124 (100 nM) with 1 ml of DMEM containing 2% FBS. After 24 hours of incubation in 5% CO2 at 37°C and 95% humidified air to allow VEGF protein secretion, the conditioned medium was collected, and 1 mM phenyl methyl sulphonyl fluoride (PMSF) was added. The supernatant was clarified by centrifugation for 5 minutes at 14,000 rpm, aliquoted, and stored at -80°C until analysis.

### Clonogenicity assay

U251 and U87MG cells were pretreated with DMSO (<0.1%) or JSI-124 (100 nM) for 2 h. The pretreated cells were throughoutly washed with serum-free medium for three times to remove all drugs. U251 (1×10^3^) and U87MG (1×10^3^) cells then were plated onto a 6-well tissue culture plate in complete medium and incubated at 37°C. Cells were allowed to grow in complete medium for 5 days. Then cells were fixed and stained with 1% Toluidine Blue in 1% borax and counted under the microscope (×50 magnification). Five random fields were counted under a light microscope at ×50 magnification.

### Endothelial cell capillary-like tube formation assay

Matrigel was thawed at 4°C, and each well of prechilled 96-well plates was coated with 30 μl matrigel and incubated at 37°C for 45 min. HUVECs (4×10^4^) were added in 200 μl ECM with various concentrations of JSI-124. After 4 h of incubation at 37°C, 5% CO2, tubular structure formation was captured under microscope and measured length of tube by using Image-Pro Plus software.

To examine the effect of JSI-124 on tumor cell–induced tube formation of HUVECs, a conditioned medium was collected from U87MG cells and used as the growth medium for HUVECs. Briefly, cells were seeded at 70% confluency; after overnight incubation, cells were treated in the presence or in the absence of JSI-124, as indicated, for 8 hours. After 8 hours, cells were washed thoroughly with phosphate-buffered saline (PBS) and further incubated in reduced serum containing DMEM for another 24 hours and collected as a conditioned medium. The conditioned medium was then used to study the in vitro tube formation assay in HUVECs, as described above.

To examine the effect of JSI-124 on VEGF-induced tube formation, HUVECs suspended in endothelial cell basal medium containing 0.5% fetal bovine serum (FBS) were seeded on a culture plate coated with growth factor–reduced Matrigel. JSI-124 was added to the cell suspension 30 minutes before plating the cells, and recombinant human VEGF_165_ (20 ng/ml) was added at the time of seeding as indicated.

### Transwell migration and invasion assay

The motility and invasion of HUVECs and GBM cells were determined using a transwell assay (Corning, Inc.) with 6.5-mm-diameter polycarbonate filters (8-μm pore size). The chambers of transwell invasion assay were coated 50% matrigel, while transwell migration assay coated no matrigel. The two chambers were coated with 0.1% gelatin for 30 min in cell incubator. The bottom transwell chambers were filled with ECM or DMEM with 0.5% FBS supplemented with or without 20ng/mL VEGF, and the top chambers were seeded 4×10^4^ cells/per well HUVECs or GBM cells in 100 μL ECM or DMEM (0.5% FBS) plus different concentrations of JSI-124. Cells were allowed to migrate for 8 h. To assay for glioblastoma cell–induced migration of endothelial cells, we performed a coculture assay using migration chambers as described by Tsujii et al [23]. Nonmigrated or noninvasive cells were removed with cotton swabs, and migrated or invasive cells were fixed with cold 4% paraformaldehyde and stained with eosine or crystal violet. Images were taken using an inverted microscope.

### Cell death detection ELISA^Plus^ assay

Cell death detection ELISA^Plus^ assay (Roche) was performed to determine apoptosis by quantification of histone-complexed DNA fragments according to the manufacturer’s instruction and absorbance was determined at 405 nm wavelength.

### Flow cytometry assay

After treating cells with various treatments, we measured apoptosis using Alexa Fluor 488 annexin V/Dead Cell Apoptosis Kit (Invitrogen, Ltd, USA) according to the manufacturer’s instructions. Briefly, cells were washed twice with cold PBS and collected and then resuspended in Annexin V binding buffer at a concentration of 5× 10^6^ cells/ml. Afterward, 1×10^6^ cells were transferred to a tube and subsequently stained with 5μl Alexa Fluor 488 annexin V and 100μg/ml propidium iodide (PI) to each 100μl of cell suspension. After incubated in the dark for 15 min at room temperature, stained cells were then analyzed by flow cytometry.

### Tumor xenograft model

Balb/c nude (nu/nu) female mice were purchased from Vital River Laboratories. U87MG cells (5×10^6^ cells in 50μl of serum-free DMEM) were inoculated subcutaneously into the right flank of five-week-old female mice after acclimated for a week. Tumor growth was measured daily with calipers. Tumor volume was calculated as (L×W^2^)/2, where L is the length in millimeters, and W is the width in millimeters. When the tumors reached a mean volume of 90 to 120 mm^3^, 12 mice were randomly assigned to JSI-124 (1 mg/kg/day, in 20% DMSO in PBS) or drug vehicle control (20% DMSO in PBS) and dosed i.p. with 100μl vehicle of drug once daily for 18 days. Tumors were dissected and frozen in liquid nitrogen or fixed in formalin.

### Statistical analysis

The data were expressed as means ±S.D. Statistical analysis was performed with the two-tailed Student’s test. The criterion for statistical significance was set at *P*<0.05.

## Results

### JSI-124 inhibits cell viability and induces apoptosis in GBM cells

**Fig 1:**
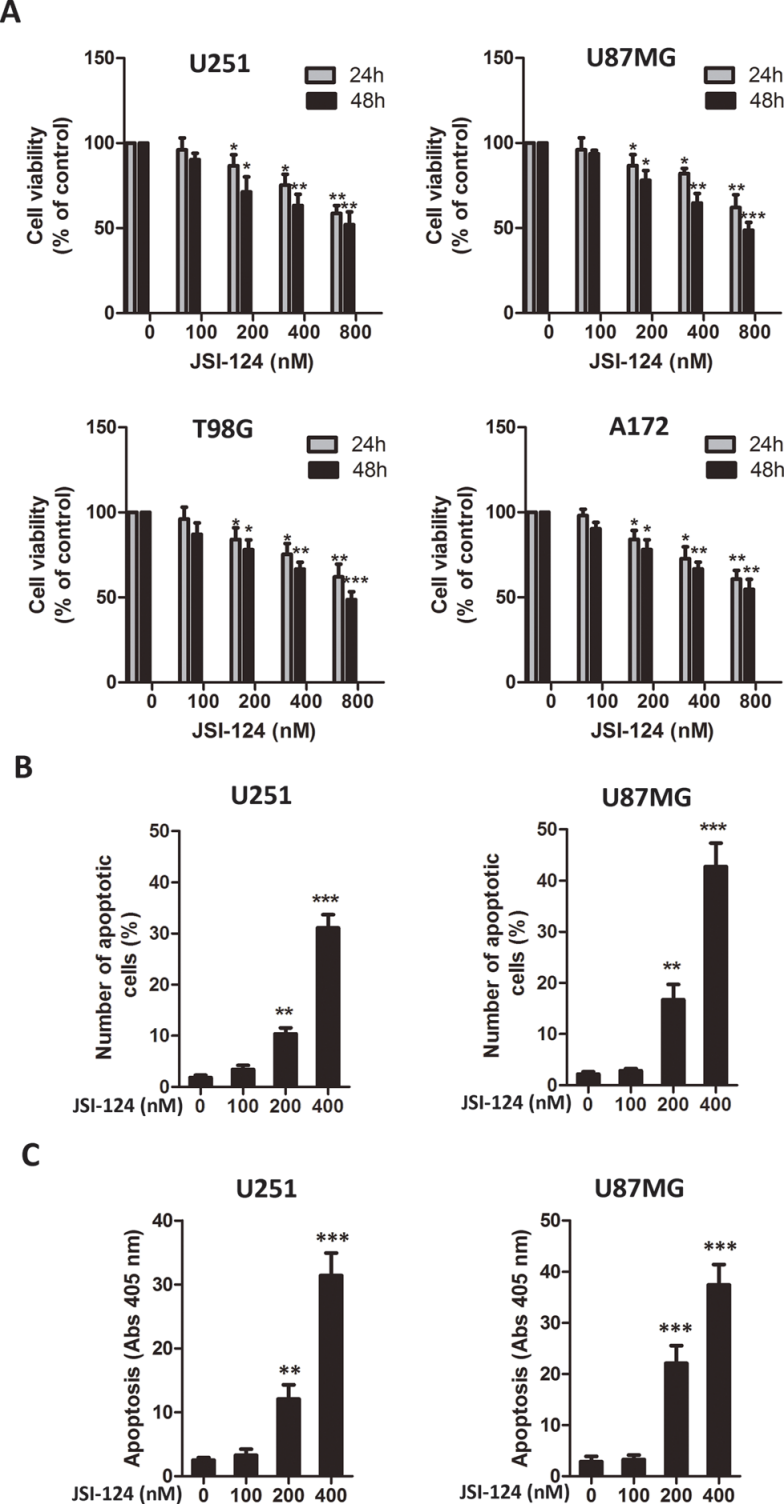
JSI-124 inhibits cell viability and induces apoptosis in GBM cells. (A) JSI-124 at high dose suppressed viability of GBM cell lines dose-dependently determined by CCK-8 assay. (B) JSI-124–mediated GBM cells apoptosis was detected by flow cytometric assay. (C) JSI-124–induced GBM cells apoptosis was measured by cell death detection ELISA^Plus^ assay. All data were expressed as mean ± SD. * *P* < 0.05; ** *P* < 0.01; *** *P* < 0.001 versus control.

To assess the effect of JSI-124 on GBM cells, we first examined several GBM cell lines treated with various doses of JSI-124 by CCK-8 assay. JSI-124 inhibited cell viability in a dose- and time-dependent manner, and significant cell viability inhibitory effect of JSI-124 was observed at concentrations more than 100nmol/L (Fig. 1A). JSI-124 exhibited anticancer potential through the induction of apoptosis in a wide variety of human tumor cell lines [19,21,22]. To verify apoptotic property on GBM cells, flow cytometry assay and cell death detection ELISA^Plus^ assay were performed. No change in apoptosis was observed at 100nM JSI-124 for 48 h, but higher concentration JSI-124 significantly induced GBM cells apoptosis dose-dependently (Fig. 1B and S1 Fig.). The similar phenomenon was observed in GBM cells determined by cell death detection ELISA^Plus^ assay (Fig. 1C).

### JSI-124 treatment at low dose inhibits invasiveness of GBM cells

**Fig 2:**
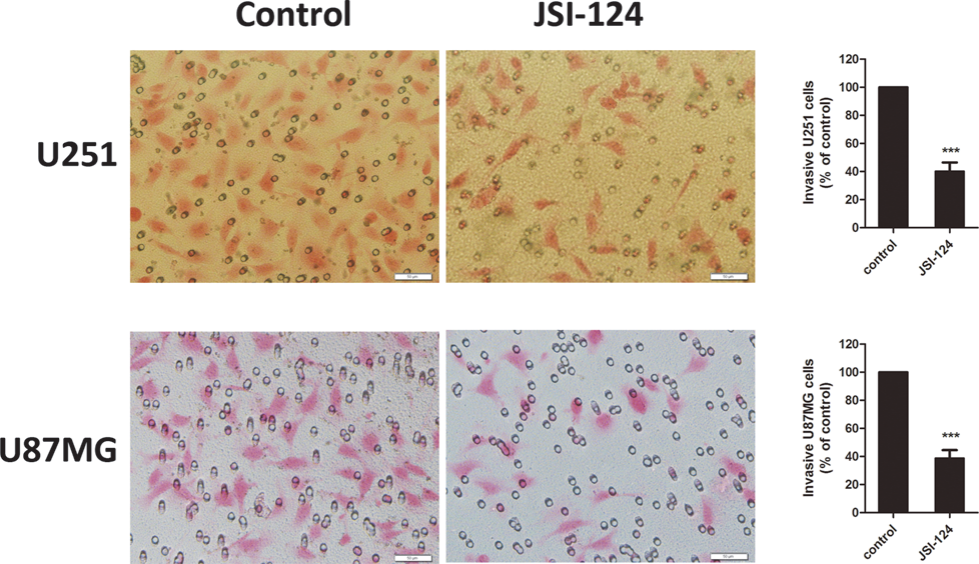
JSI-124 treatment at low dose inhibits invasiveness of GBM cells. Effect of JSI-124 at 100 nM on GBM cells invasiveness performed by transwell invasion analysis. All data were expressed as mean ± SD (n = 10 fields). ***P< 0.001, versus control.

We next examined if the effect of JSI-124 at low dose on invasiveness of GBM cells. As show in Fig. 2, transwell invasion assay revealed 100 nM JSI-124 treatment significantly inhibited GBM cells invasion ability at 8 h.

### Exposure of GBM cells to JSI-124 at low dose reduces clonogenicity

**Fig 3:**
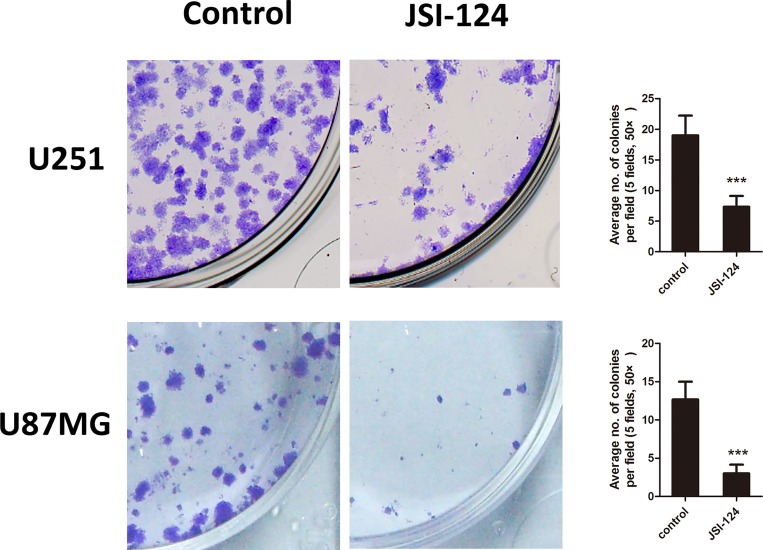
Exposure of GBM cells to JSI-124 at low dose reduces clonogenicity. Number of multicellular colonies was reduced by JSI-124 at 100 nM treatment for 2 h. Colonies with >10 cells per colony were counted. The average number of established colonies per field was presented as mean ± SD (n = 5 fields). ***P< 0.01, versus control.

We further examined if low-dose JSI-124 would be sufficient to prevent GBM cells tumorigenicity. Using an *in vitro* clonogenic assay, we found that just brief exposure of GBM cells to JSI-124 (100 nM) for 2 h was sufficient to significantly reduce the number of tumor colonies of U251 and U87MG cells (Fig. 3), suggesting that JSI-124 may inhibit GBM tumorigenicity.

### JSI-124 inhibits VEGF expression in GBM cells

**Fig 4:**
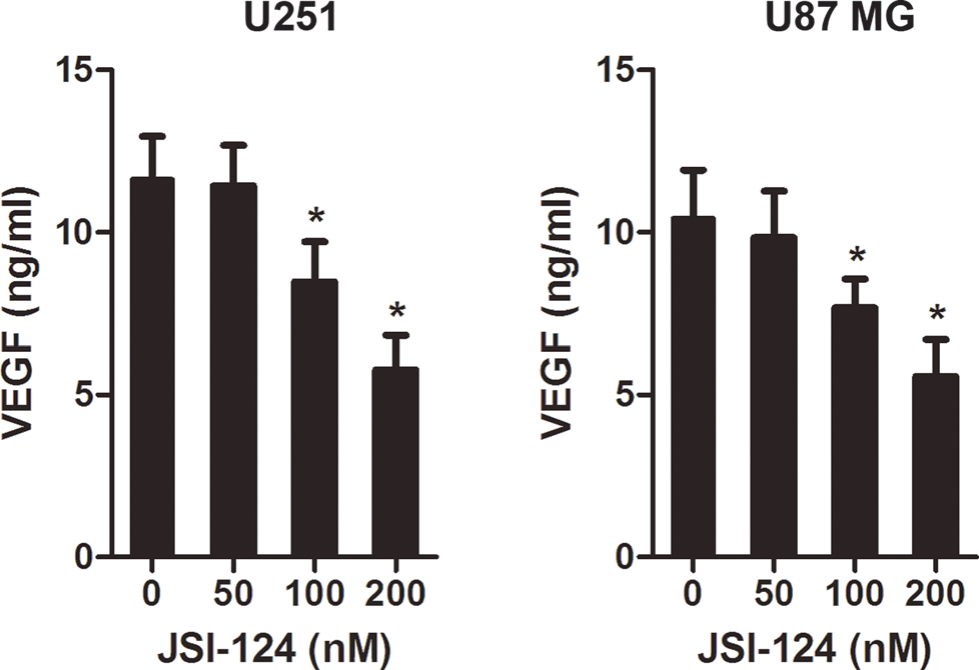
JSI-124 inhibits VEGF expression in GBM cells. Cells were exposed to different doses of JSI-124 for 24 hours and VEGF protein released by GBM cells into the conditioned medium was measured by ELISA kit. All data were expressed as mean ± SD. * *P* < 0.05, versus control.

VEGF is a critical factor in new blood vessel formation [6,24]. In a tumor microenvironment, cancer cells secrete a high level of VEGF that binds to receptors on surrounding endothelial cells, promoting endothelial cell migration, proliferation, and differentiation, as well as tube formation [25,26]. In this experiment, we measure the effect of JSI-124 on the VEGF level in GBM cells at different concentrations by ELISA. The treatment of JSI-124 at 100nM for 24 hours markedly reduced the secretion of VEGF by GBM cells (Fig. 4).

### JSI-124 inhibites the U87MG cells–induced tube formation of HUVECs

**Fig 5:**
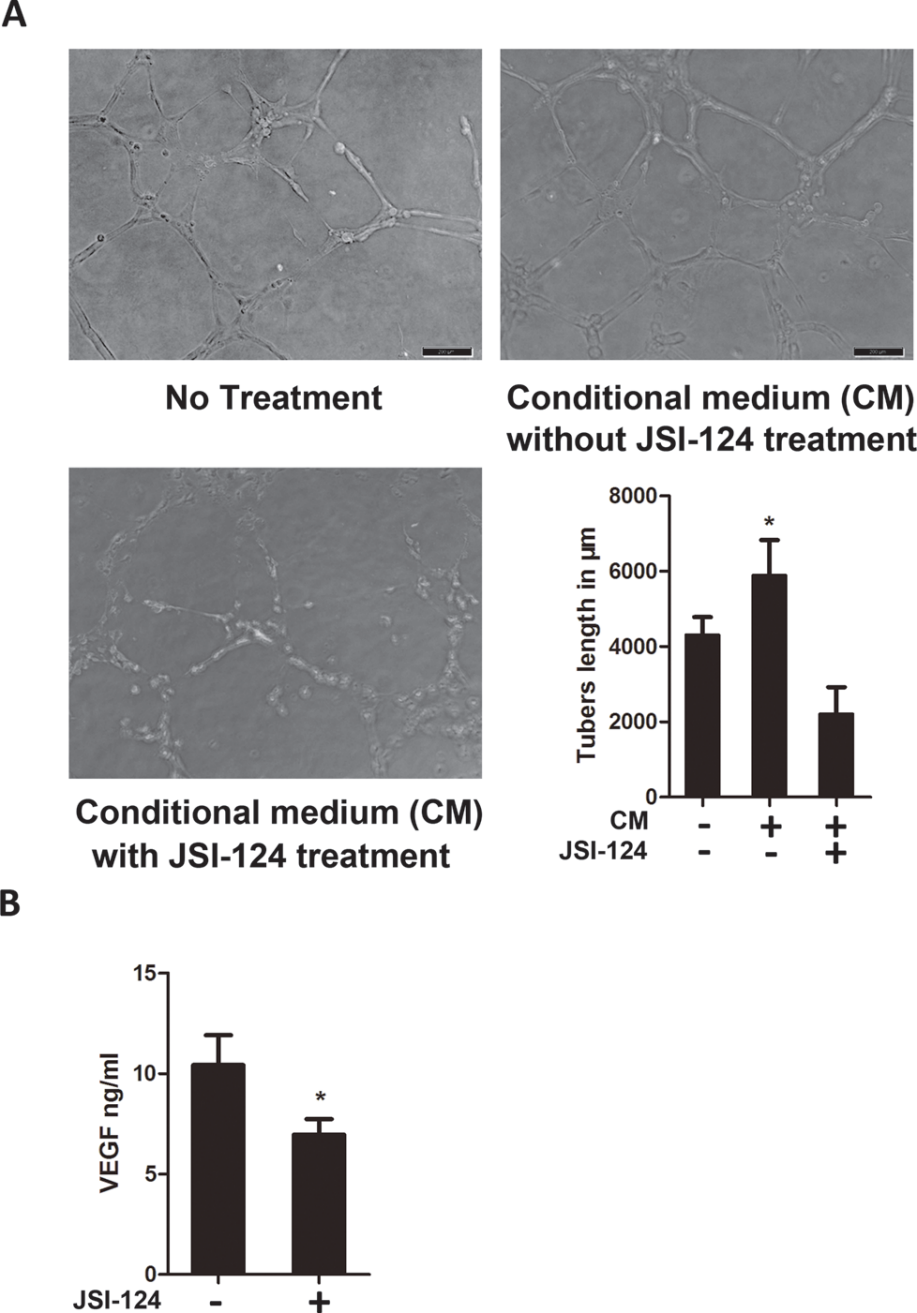
JSI-124 inhibites the U87MG cells–induced tube formation of HUVECs. (A) U87MG cells were treated with or without JSI-124 for 8 hours and then incubated with fresh media without JSI-124 for 24 hours, followed by the collection of conditioned media (CM). (B) VEGF level was analyzed in the conditioned medium by ELISA.

In this experiment, we used conditioned media from U87MG cells treated with or without JSI-124 to determine whether GBM cells could induce the capillary formation of HUVECs and examine the effect of on this event. It is clearly seen that the conditioned medium (without JSI-124) from U87MG cells induced capillary formation of HUVECs. However, the conditioned medium (with JSI-124) from U87MG cells produced a complete prevention of tube formation (Fig. 5A). We also measured the VEGF level in the conditioned medium obtained from U87MG cells, a marked inhibition of VEGF production was noticed in the presence of JSI-124 (Fig. 5B). These results demonstrated that the capillary formation of HUVECs was augmented by GBM cells and that this activity was downregulated after JSI-124 treatment.

### JSI-124 inhibites the U87MG cells–induced migration of HUVECs

**Fig 6:**
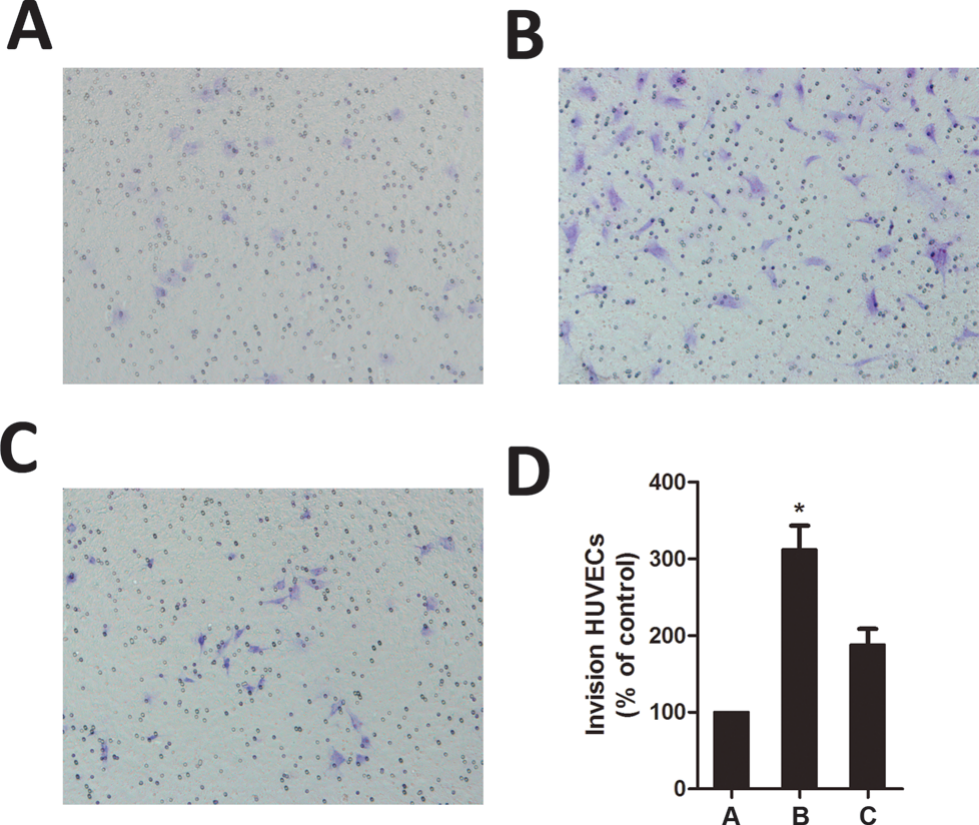
JSI-124 inhibites the U87MG cells–induced migration of HUVECs. U87MG cells were cultured in the well (lower chamber). Cells were then treated with or without JSI-124 for 8 hours. After treatment, the medium from the lower chamber was replaced with fresh DMEM without JSI-124, and the insert containing a monolayer of HUVECs in DMEM was then placed into the well. After 8 hours, migrated cells were photographed and counted. (A) Schematic diagram of coculture assay. (B) Control. HUVECs were seeded onto the upper chamber, whereas the bottom chamber contains only DMEM without U87MG cells. (C) HUVECs cocultured with U87MG cells untreated with JSI-124. (D) HUVECs cocultured with U87MG cells pretreated with 100 nM. All data were expressed as mean ± SD. * *P* < 0.05, versus control.

We further examined the effect of JSI-124 on HUVECs migration induced by GBM cells using a coculture assay. The HUVECs cocultured with U87MG cells (Fig. 6B) triggered pronounced migration compared with HUVECs alone (Fig. 6A), while this increased migration of HUVECs was prevented significantly when U87MG cells were treated with JSI-124(Fig. 6C). These results revealed an increased migration of endothelial cells when cocultured with GBM cells, and this migration was severely inhibited when GBM cells were treated with JSI-124.

### JSI-124 inhibits cell viability and induces apoptosis in HUVECs

**Fig 7:**
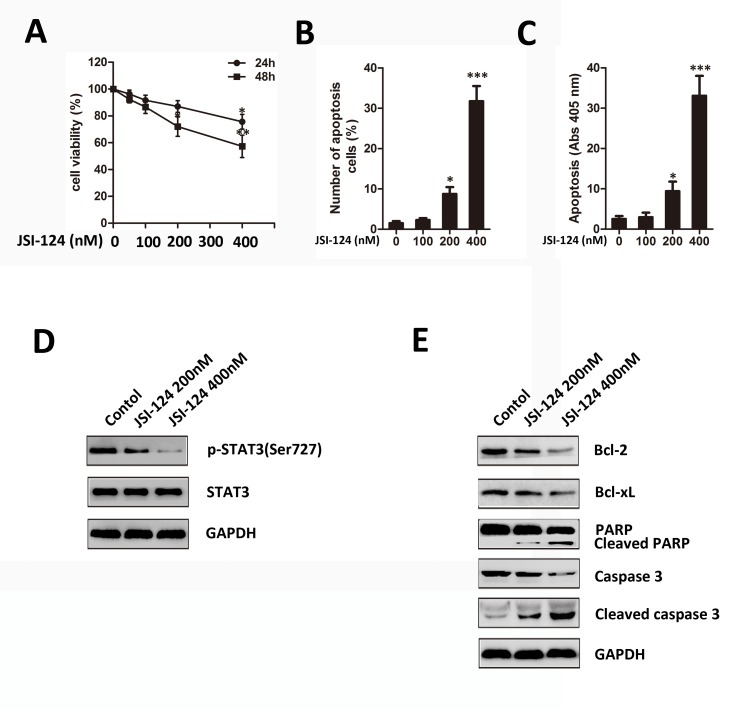
JSI-124 inhibits cell viability and induces apoptosis in HUVECs. (A) Effect of JSI-124 on HUVECs under normal culture condition. HUVECs (5×10^3^/ well) were treated with different concentrations of JSI-124 for 24 and 48 h. (B) JSI-124 induced HUVECs apoptosis dose-dependently detected by flow cytometric assay. (C) JSI-124 induced GBM cells apoptosis dose-dependently detected by cell death detection ELISA^Plus^ assay. All data were expressed as mean ± SD. * *P* < 0.05; ** *P* < 0.01; *** *P* < 0.01 versus control. (D) and (E) JSI-124 downregulated the level of p-STAT3, decreased the expression of Bcl-2 and Bcl-xL, and induced cleavage of caspase-3 and PARP in HUVECs in a dose-dependent manner by western blot analysis. GAPDH was shown as loading control.

To systematically address the inhibitory activity of JSI-124 on HUVECs growth, we first evaluated the cell viability by CCK-8 assay. Treatment with JSI-124 resulted in growth inhibition in HUVECs in a dose-dependent manner (Fig. 7A). We then examined the apoptotic effects of JSI-124 by flow cytometry and cell death detection ELISA^Plus^ assay. We found that the proportion of apoptotic cells was significantly increased in a dose-dependent manner in JSI-124-treated HUVECs for 48h (Fig. 7B and S2 Fig.). Similar results were found in HUVECs performed by cell death detection ELISA^Plus^ assay (Fig. 7C). Moreover, JSI-124 treatment for 48h down-regulated the expression of p-STAT3 (Ser727) (Fig. 7D). In addition, Bcl-2 and Bcl-xL significantly decreased as well as clear cleavages of PARP and caspase-3 occurred dose-dependently (Fig. 7E).

### JSI-124 inhibits VEGF-induced migration, invasion and tubular structure formation of HUVECs

**Fig 8:**
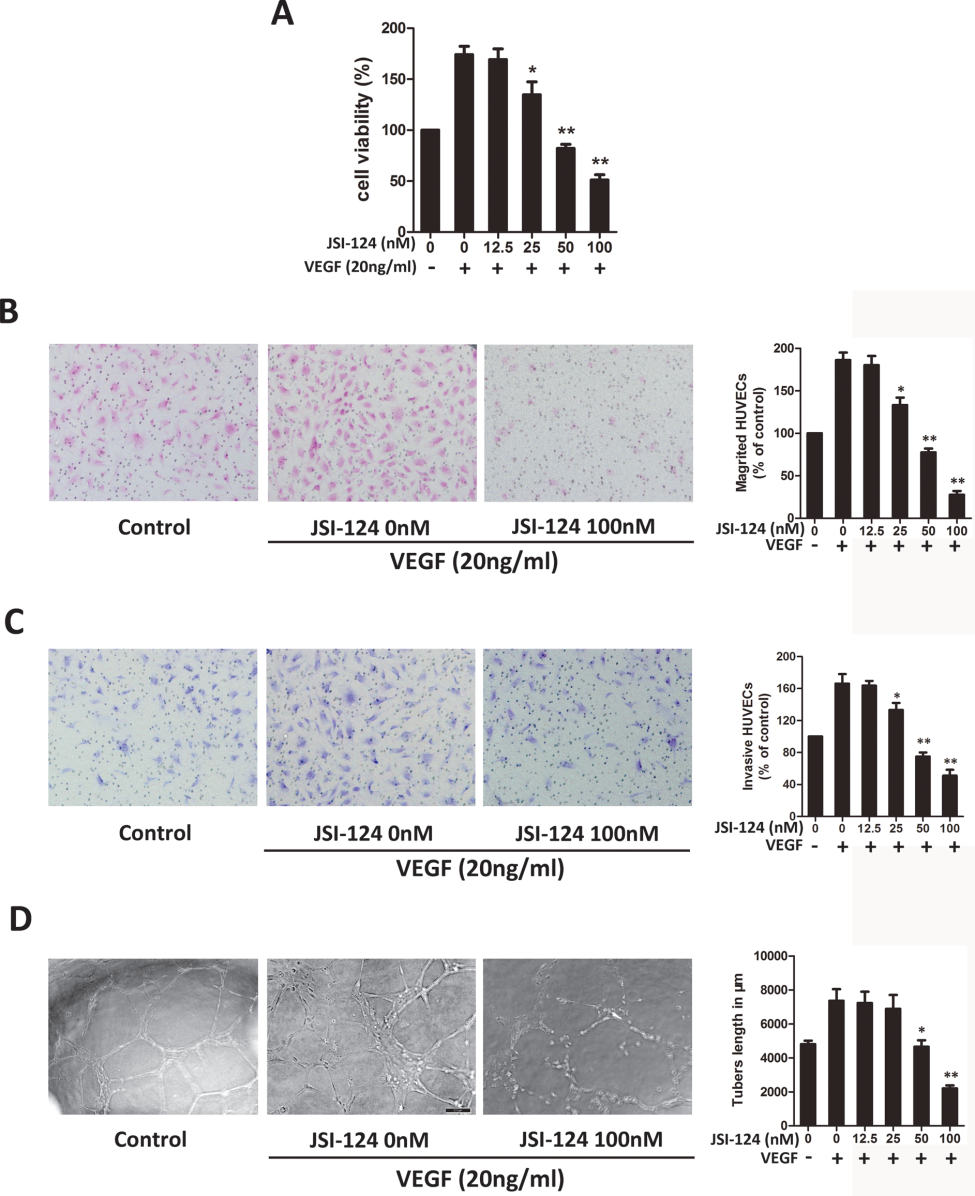
JSI-124 inhibits VEGF-induced migration, invasion and tubular structure formation of endothelial cells. (A) Effect of JSI-124 on VEGF-induced HUVECs proliferation determined by CCK-8 assay. HUVECs (5×10^3^/ per well) were starved with 0.1% FBS medium and then treated with or without VEGF (20 ng/mL) and different concentrations of JSI-124 for 24 h. (B) JSI-124 remarkably inhibited VEGF-induced endothelial cells migration measured by transwell migration assay. HUVECs were seeded in the upper chamber of transwell (coated no matrigel) and treated with different concentrations of JSI-124. The bottom chamber was filled with ECM supplemented with VEGF. After about 8 h, the migrated HUVECs passed through the membrane were quantified. (C) JSI-124 strongly suppressed VEGF-induced endothelial cells invasion measured by transwell invasion assay. HUVECs were seeded in the upper chamber of transwell (coated with 50% matrigel) and treated with different concentrations of JSI-124. The bottom chamber was filled with ECM supplemented with VEGF. After about 8 h, the migrated HUVECs passed through the membrane were quantified. (D) JSI-124 inhibits VEGF-induced tube formation of HUVECs determined by capillary-like tube formation assay. Cells (4×10^4^/ well) were placed in the 96-well plates coated with matrigel. After 4 h of incubation, tubular structure formation was captured under microscope. All data were expressed as mean ± SD. * *P* < 0.05; ** *P* < 0.01 versus VEGF alone.

To assess the antiangiogenic effect of JSI-124 on endothelial cells, cell viability of HUVECs was determined by CCK-8 assay. As shown in Fig. 8A, the proliferation of HUVECs stimulated by VEGF (20ng/ml) was markedly decreased after JSI-124 treatment ranging from 25 to 100nmol/L for 24 h indicating extracellular VEGF acted as a strong stimulus for HUVECs proliferation. Cell migration is an essential step for endothelial cell to form blood vessels in angiogenesis [27]. Next transwell migration assay and transwell invasion assay were utilized to investigate the inhibitory effects of JSI-124 on the motility of HUVECs. The results showed that JSI-124 inhibited VEGF-induced HUVEC migration (Fig. 8B) and invasion (Fig. 8C) in a dose-dependent manner.

The maturation of migrated endothelial cells into a capillary tube is a critical step during angiogenesis [28]. To examine the potential effects of JSI-124 on the tubular structure formation, we conducted two-dimensional matrigel assays and examined JSI-124 effect on tubular structure formation in HUVECs. VEGF significantly enhanced the tubular network formation compared to HUVECs seeded on matrigel under 0.5% serum in ECM alone; however, treatment with JSI-124 strongly inhibited the VEGF-stimulated tubular network formation, and tube length measurement showed that JSI-124 treatment inhibited the tube length dose-dependently (Fig. 8D). Overall, these results indicated that JSI-124 could suppress VEGF-induced angiogenesis by inhibiting migration, invasion, and tube formation of HUVECs.

### JSI-124 inhibits activation of VEGFR2 and JAK2/STAT3 signaling induced by VEGF in HUVECs

**Fig 9:**
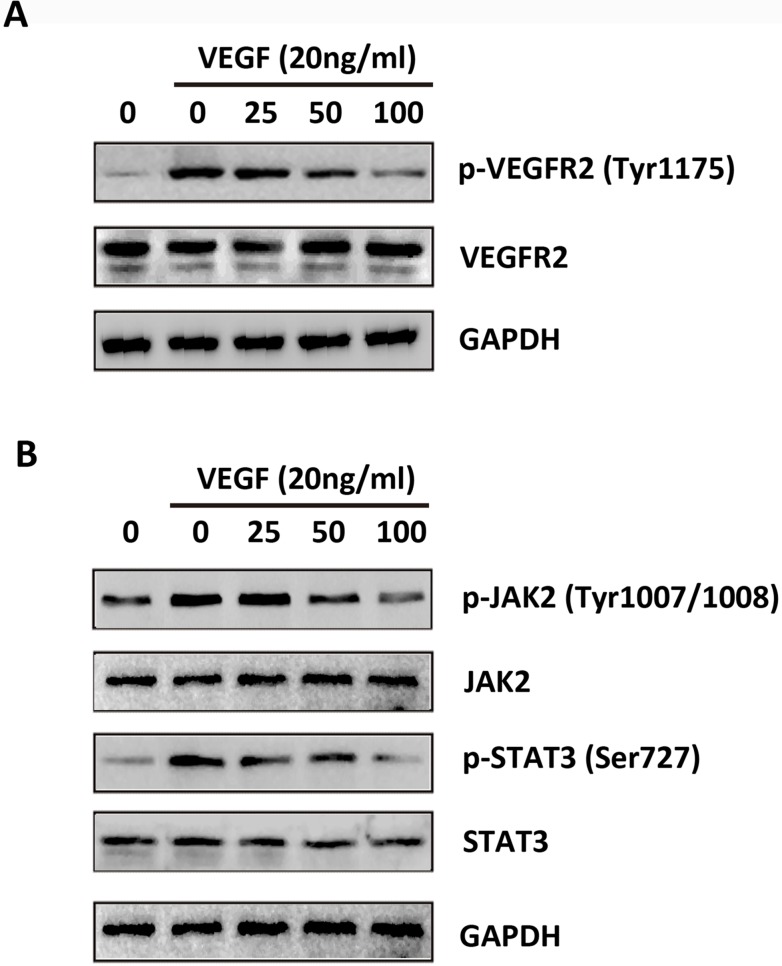
JSI-124 inhibits activation of VEGFR2 and JAK2/STAT3 signaling induced by VEGF in HUVECs. (A) JSI-124 suppressed the activation of VEGFR2 induced by VEGF in HUVECs in a dose-dependent manner by western blot analysis. (B) Inhibition p-VEGFR2 by JSI-124 resulted in an inhibition of JAK2/STAT3 pathway activation triggered by VEGF in HUVECs dose-dependently by western blot analysis. GAPDH was shown as loading control.

VEGFR2 activation is responsible for endothelial cell migration and proliferation [29]. We examined the effects of JSI-124 on phosphorylation of VEGFR2 to determine its inhibitory effect on VEGFR2-mediated signaling pathways in HUVECs. We found that VEGFR2 was strongly phosphorylated by exogenous VEGF to HUVECs, while treatment with JSI-124 significantly blocked VEGF-induced phosphorylation of VEGFR2 in a dose-dependent manner without affecting overall VEGFR2 expression levels (Fig. 9A). Previous studies suggested that VEGF triggered the activation of STAT3 signaling in HUVECs [30–32]. The effects of JSI-124 on the JAK2/STAT3 signaling were determined by using western blot analysis. In addition, JSI-124 significantly suppressed the phosphorylation of JAK2 (Tyr1007/1008) and STAT3 (Ser727) stimulated by VEGF in a dose-dependent manner (Fig. 9B). These results provide evidence that JSI-124 blocked angiogenesis by targeting JAK2/STAT3 signaling pathway.

### JSI-124 inhibits tumor growth and angiogenesis in U87MG cells xenograft model

**Fig 10:**
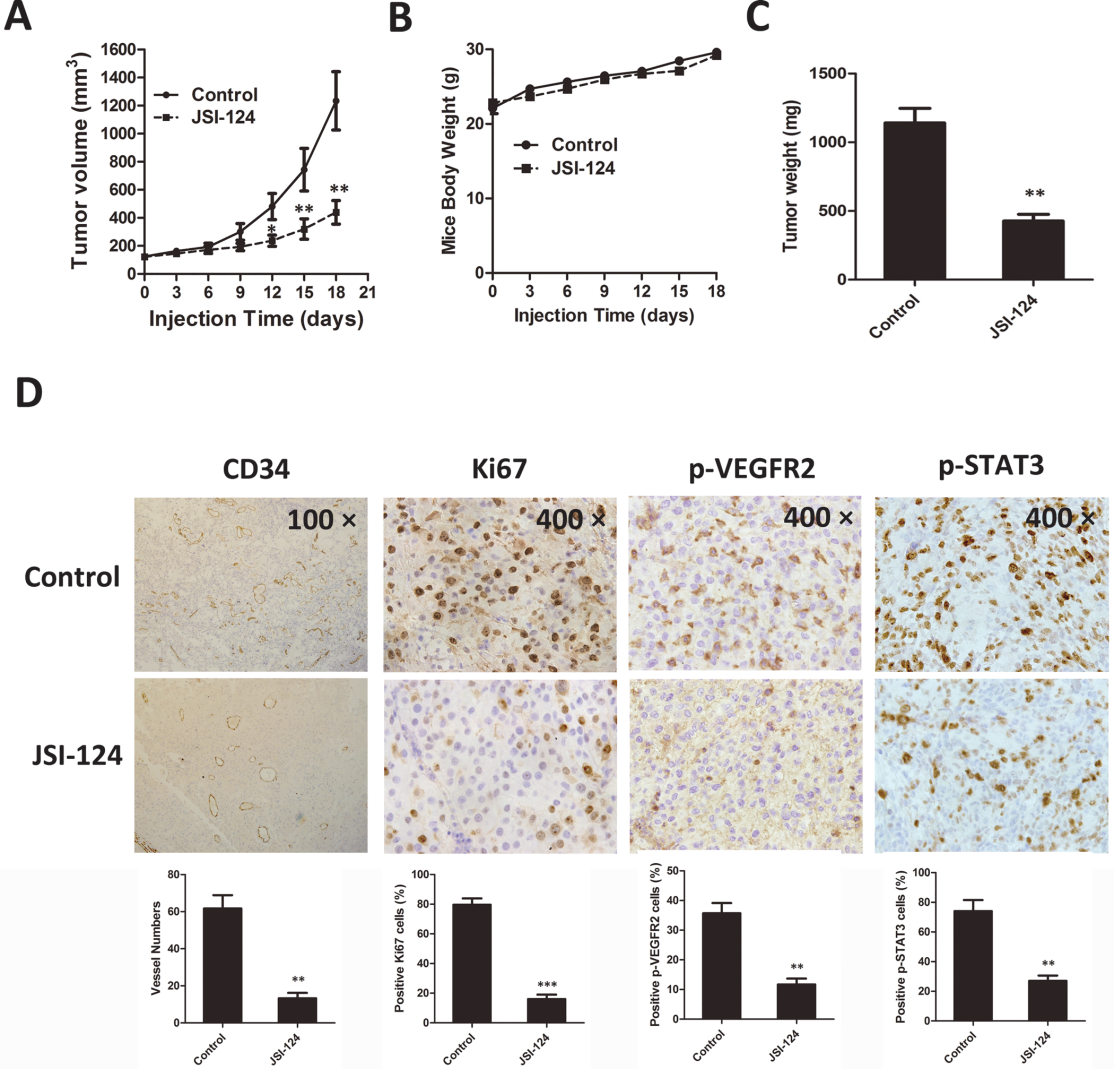
JSI-124 inhibits tumor growth and angiogenesis in U87MG cells xenograft model. U87MG cells (5×10^6^ cells per mouse) were inoculated subcutaneously into the right flank of 5-wk-old female Babl/c nude mice. Tumors reached a mean volume of 90 to 120 mm^3^, mice were randomly assigned to JSI-124 (1 mg/kg/day, in 20% DMSO in PBS) or drug vehicle control (20% DMSO in PBS) and dosed i.p. with 100μl vehicle of drug once daily for 18 days. (A) JSI-124 significantly inhibited tumor growth measured by tumor volume. (B) JSI-124 had little toxicity in the amount exposed measured by mice body weight. (C) JSI-124 strongly suppressed solid tumors. (D) Immunohistochemical staining result of CD34, Ki67, p-VEGFR2 and p-STAT3 on tumor sections. Data were presented as means ± SD. * *P* < 0.05; ** *P* < 0.01 versus control group.

To evaluate the effects of JSI-124 on tumor growth and tumor angiogenesis *in vivo*, we further constructed a series of therapeutic experiments using U87MG cells xenograft mouse model. No major side effects were noted throughout the study. We found that intraperitoneal administration of JSI-124 (1mg/kg/d, 18 days) markedly inhibited tumor volumn and tumor weight as compared with the counterparts treated with DMSO. The average tumor volume of solid tumors in JSI-124-treated mice was 432 mm^3^ (±90), as compared with 1210 mm^3^ (±230) for control group (Fig. 10A). Moreover, there was no effect on body weight of mice (Fig. 10B). The average tumor weights at study termination were 1,450 mg (±285) and 446 mg (±96) in control and JSI-124 group, respectively (Fig. 10C).

We further showed the immunohistochemical staining results of Ki67, CD34, p-VEGFR2 and p-STAT3, on tumor sections. As shown in Fig. 10D, intraperitoneal injections of JSI-124 resulted in pronounced decrease in tumor cell proliferation and CD34-positive endothelial cells, and marked reduction expression of p-VEGFR2 and p-STAT3 in solid tumors.

## Discussion

Glioblastoma, the most common brain tumor and are associated with high invasion, is resistant to conventional treatment methods and have poor prognosis mainly due to uncontrolled angiogenesis and resultant high tumor mass [9]. The current radioactive and chemotherapeutic regimens also kill non-specifically functional and non-cancerous cells in the brain, which might adversely impair different functions of the body. To prevent this highly invasive cancer from occurring, preventive approaches are highly desirable. In this study, we demonstrated for the first time that JSI-124 reduced invasiveness and tumorigenicity of GBM cells at low dose. JSI-124 effectively inhibited VEGF expression in GBM cells. In addition, JSI-124 prevented U87MG cell–mediated capillary formation of HUVECs and the migration of HUVECs when cultured alone or cocultured with U87MG cells. Moreover, JSI-124 significantly inhibited angiogenesis including human endothelial cell proliferation, migration, invasion and capillary structure formation in a concentration-dependent manner as well as the stimulative effects of human endothelial cell death in response to VEGF *in vitro*. Immunohistochemistry analysis showed that the expression of CD34, Ki67, p-STAT3 and p-VEGFR2 protein in xenografts was remarkably decreased. Taken together, our results suggested that JSI-124 may be a potent chemopreventive agent for GBM with anti-invasion and anti-angiogenisitic activities.

JSI-124 has been reported to inhibit the proliferative activity of several types of cancer cells associated with apoptotic cell death mediated via the inhibition of the constitutively activated STAT3 signaling pathway [19,21,33,34]. Consistent with these findings, our results showed that JSI-124 induced apoptosis dose-dependently in GBM at higher than 100 nM. GBMs are highly infiltrative into brain preventing surgical cure even with heroic resections [35,36], invading tumor cells appear to be particularly resistant to cytotoxic therapy and are often protected by an intact neurovascular unit [37]. Our data provided the evidence that invasiveness of GBM cells was significantly inhibited after low-dose JSI-124 treatment Moreover, we showed that brief exposure to low-dose JSI-124 can reduce the clonogenicity of GBM cells *in vitro*.

The importance of tumor angiogenesis in cancer progression is underscored by the fact that it is an important target for the development of anticancer therapies based on the inhibition of angiogenesis, and antiangiogenic therapy is now considered as the forth strategy to treat cancer [8,38,39]. Angiogenesis is a complex multistep process that involves endothelial cell proliferation, migration, and tube formation triggered by specific growth factors in tumor microenvironment [39]. Cancer cells produce numerous angiogenic factors, including VEGF, FGF, EGF, PDGF etc, which play a pivotal role in the development of tumor angiogenesis by stimulating endothelial cell proliferation, migration, and capillary tube formation [40–42]. Among all angiogenic factors, VEGF is identified as a key mediator of angiogenesis [6]. Glioblastomas secrete a very large quantity of VEGF protein into the surrounding microenvironment, thereby allowing endothelial cell proliferation, migration, and tube formation [43,44]. In this study, we found that the basal VEGF levels in U87MG and U251 cells were significantly high and that JSI-124 dose-dependently downregulated protein levels of VEGF. Using a coculture assay, we examined the effect of this inhibitor on glioblastoma cell–induced migration and on the tube formation of endothelial cells. Treatment with JSI-124 decreased VEGF secretion by U87MG cells and thereby reduced endothelial cell migration and tube formation.

Moreover, we showed that JSI-124 blocked the proliferation, capillary formation, and migration of endothelial cells, all of which are critical steps for angiogenesis. We further showed that induction of apoptosis in endothelial cells by JSI-124 was due to the downregulation of Bcl-2 and Bcl-xL, both of which are apoptotic proteins overexpressed in endothelial cells and associated with resistance to apoptosis. Overexpression of VEGF and VEGF receptors correlates with increased microvessel density, proliferation, and tumor growth rate, which lead to poor patient prognosis in a variety of malignancies [45,46]. We provided first evidence that JSI-124 effectively abrogated VEGF-induced HUVECs proliferation, invasion, migration, and capillary-like structures formation *in vitro*. Phosphorylation of VEGFR2 is critical for VEGF-mediated microvascular permeability, endothelial cell proliferation, invasion and migration [47–49]. In the present study, we found that JSI-124 blocked the activity of VEGFR2 (Tyr^1175^) by down-regulation of VEGF-induced phosphorylation of VEGFR-2 expression. A close association between STAT3 activation and glioma growth and vascularization has been reported previously [11,12,50], and activation of STAT3 has been directly correlated with VEGF production [13]. Our present investigation showed that the expression of VEGF was dose-dependently suppressed by JSI-124 via STAT3 (Ser^727^) inhibition in HUVECs. STAT3 is principally activated by nonreceptor tyrosine kinase JAK2 [51]. Our results showed that phosphorylation of JAK2 (Tyr^1007/1008^) was dose-dependently blocked by JSI-124 in HUVECs, indicating that the direct effects of JSI-124 on angiogenesis might be through inhibiting the VEGFR2/STAT3 signaling pathway.

To evaluate the antitumor activity of JSI-124 *in vivo*, Babl/c nude mice transplanted with U87MG cells were treated with JSI-124. We found that JSI-124 (1 mg/kg/day) significantly suppressed tumor volume and tumor weight without any side effects on mice, and remarkably reduced neovascularization accompanied by down-regulation expression of p-STAT3 and p-VEGFR2. In addition to the effective antiangiogenesis of JSI-124 *in vitro*, we presume that JSI-124 suppresses GBM growth *in vivo* through not only directly tumor cell proliferation inhibition but also tumor angiogenesis suppression.

Taken together, our results indicate that JSI-24 may be an effective preventive agent for GBM with anti-invasion and anti-angiogenesitic activity. It is hoped that in the future, patients harboring GBM can be offered an affordable preventive agent to reduce the incidence of GBM.

## Supporting Information

S1 Fig JSI-124 induced GBM cells apoptosis dose-dependently detected by flow cytometric assay. (TIF)

S2 Fig JSI-124 induced HUVECs apoptosis dose-dependently detected by flow cytometric assay. (TIF)

## References

[1] Louis DN, Ohgaki H, Wiestler OD, Cavenee WK, Burger PC, et al (2007) The 2007 WHO classification of tumours of the central nervous system. Acta Neuropathol 114: 97–109. 17618441 10.1007/s00401-007-0243-4 PMC1929165

[2] Fan S, Sun Z, Jiang D, Dai C, Ma Y, et al (2010) BmKCT toxin inhibits glioma proliferation and tumor metastasis. Cancer Lett 291: 158–166. 10.1016/j.canlet.2009.10.011 19906483

[3] Louis DN, Pomeroy SL, Cairncross JG (2002) Focus on central nervous system neoplasia. Cancer Cell 1: 125–128. 12086870 10.1016/s1535-6108(02)00040-5

[4] Omuro AM, Faivre S, Raymond E (2007) Lessons learned in the development of targeted therapy for malignant gliomas. Mol Cancer Ther 6: 1909–1919. 17620423 10.1158/1535-7163.MCT-07-0047

[5] Skobe M, Rockwell P, Goldstein N, Vosseler S, Fusenig NE (1997) Halting angiogenesis suppresses carcinoma cell invasion. Nat Med 3: 1222–1227. 9359696 10.1038/nm1197-1222

[6] Folkman J (1995) Angiogenesis in cancer, vascular, rheumatoid and other disease. Nat Med 1: 27–31. 7584949 10.1038/nm0195-27

[7] Hanahan D, Weinberg RA (2000) The hallmarks of cancer. Cell 100: 57–70. 10647931 10.1016/s0092-8674(00)81683-9

[8] Cook KM, Figg WD (2010) Angiogenesis inhibitors: current strategies and future prospects. CA Cancer J Clin 60: 222–243. 10.3322/caac.20075 20554717 PMC2919227

[9] Onishi M, Ichikawa T, Kurozumi K, Date I (2011) Angiogenesis and invasion in glioma. Brain Tumor Pathol 28: 13–24. 10.1007/s10014-010-0007-z 21221826

[10] Jain RK, di Tomaso E, Duda DG, Loeffler JS, Sorensen AG, et al (2007) Angiogenesis in brain tumours. Nat Rev Neurosci 8: 610–622. 17643088 10.1038/nrn2175

[11] Doucette TA, Kong LY, Yang Y, Ferguson SD, Yang J, et al (2012) Signal transducer and activator of transcription 3 promotes angiogenesis and drives malignant progression in glioma. Neuro Oncol 14: 1136–1145. 10.1093/neuonc/nos139 22753228 PMC3424209

[12] de Groot J, Liang J, Kong LY, Wei J, Piao Y, et al (2012) Modulating antiangiogenic resistance by inhibiting the signal transducer and activator of transcription 3 pathway in glioblastoma. Oncotarget 3: 1036–1048. 23013619 10.18632/oncotarget.663 PMC3660053

[13] Chen Z, Han ZC (2008) STAT3: a critical transcription activator in angiogenesis. Med Res Rev 28: 185–200. 17457812 10.1002/med.20101

[14] Jung JE, Lee HG, Cho IH, Chung DH, Yoon SH, et al (2005) STAT3 is a potential modulator of HIF-1-mediated VEGF expression in human renal carcinoma cells. FASEB J 19: 1296–1298. 15919761 10.1096/fj.04-3099fje

[15] Buettner R, Mora LB, Jove R (2002) Activated STAT signaling in human tumors provides novel molecular targets for therapeutic intervention. Clin Cancer Res 8: 945–954. 11948098

[16] Gamero AM, Young HA, Wiltrout RH (2004) Inactivation of Stat3 in tumor cells: releasing a brake on immune responses against cancer? Cancer Cell 5: 111–112. 14998485 10.1016/s1535-6108(04)00028-5

[17] Kim MJ, Nam HJ, Kim HP, Han SW, Im SA, et al (2013) OPB-31121, a novel small molecular inhibitor, disrupts the JAK2/STAT3 pathway and exhibits an antitumor activity in gastric cancer cells. Cancer Lett 335: 145–152. 10.1016/j.canlet.2013.02.010 23402820

[18] Pathak AK, Bhutani M, Nair AS, Ahn KS, Chakraborty A, et al (2007) Ursolic acid inhibits STAT3 activation pathway leading to suppression of proliferation and chemosensitization of human multiple myeloma cells. Mol Cancer Res 5: 943–955. 17855663 10.1158/1541-7786.MCR-06-0348

[19] Su Y, Li G, Zhang X, Gu J, Zhang C, et al (2008) JSI-124 inhibits glioblastoma multiforme cell proliferation through G(2)/M cell cycle arrest and apoptosis augment. Cancer Biol Ther 7: 1243–1249. 18487947 10.4161/cbt.7.8.6263

[20] Heath VL, Bicknell R (2009) Anticancer strategies involving the vasculature. Nat Rev Clin Oncol 6: 395–404. 10.1038/nrclinonc.2009.52 19424102

[21] Blaskovich MA, Sun J, Cantor A, Turkson J, Jove R, et al (2003) Discovery of JSI-124 (cucurbitacin I), a selective Janus kinase/signal transducer and activator of transcription 3 signaling pathway inhibitor with potent antitumor activity against human and murine cancer cells in mice. Cancer Res 63: 1270–1279. 12649187

[22] Jing N, Tweardy DJ (2005) Targeting Stat3 in cancer therapy. Anticancer Drugs 16: 601–607. 15930886 10.1097/00001813-200507000-00002

[23] Tsujii M, Kawano S, Tsuji S, Sawaoka H, Hori M, et al (1998) Cyclooxygenase regulates angiogenesis induced by colon cancer cells. Cell 93: 705–716. 9630216 10.1016/s0092-8674(00)81433-6

[24] Ferrara N (1999) Molecular and biological properties of vascular endothelial growth factor. J Mol Med (Berl) 77: 527–543. 10494799 10.1007/s001099900019

[25] Grunstein J, Roberts WG, Mathieu-Costello O, Hanahan D, Johnson RS (1999) Tumor-derived expression of vascular endothelial growth factor is a critical factor in tumor expansion and vascular function. Cancer Res 59: 1592–1598. 10197634

[26] Holash J, Maisonpierre PC, Compton D, Boland P, Alexander CR, et al (1999) Vessel cooption, regression, and growth in tumors mediated by angiopoietins and VEGF. Science 284: 1994–1998. 10373119 10.1126/science.284.5422.1994

[27] Shibuya M (2006) Vascular endothelial growth factor (VEGF)-Receptor2: its biological functions, major signaling pathway, and specific ligand VEGF-E. Endothelium 13: 63–69. 16728325 10.1080/10623320600697955

[28] Patan S (2004) Vasculogenesis and angiogenesis. Cancer Treat Res 117: 3–32. 15015550 10.1007/978-1-4419-8871-3_1

[29] Ferrara N, Gerber HP, LeCouter J (2003) The biology of VEGF and its receptors. Nat Med 9: 669–676. 12778165 10.1038/nm0603-669

[30] Chen J, Wang J, Lin L, He L, Wu Y, et al (2012) Inhibition of STAT3 signaling pathway by nitidine chloride suppressed the angiogenesis and growth of human gastric cancer. Mol Cancer Ther 11: 277–287. 10.1158/1535-7163.MCT-11-0648 22203730

[31] Kandala PK, Srivastava SK (2012) Diindolylmethane suppresses ovarian cancer growth and potentiates the effect of cisplatin in tumor mouse model by targeting signal transducer and activator of transcription 3 (STAT3). BMC Med 10: 9 10.1186/1741-7015-10-9 22280969 PMC3298725

[32] Lu J, Zhang K, Nam S, Anderson RA, Jove R, et al (2010) Novel angiogenesis inhibitory activity in cinnamon extract blocks VEGFR2 kinase and downstream signaling. Carcinogenesis 31: 481–488. 10.1093/carcin/bgp292 19969552 PMC3105590

[33] Ishdorj G, Johnston JB, Gibson SB (2010) Inhibition of constitutive activation of STAT3 by curcurbitacin-I (JSI-124) sensitized human B-leukemia cells to apoptosis. Mol Cancer Ther 9: 3302–3314. 10.1158/1535-7163.MCT-10-0550 21159613

[34] Yuan G, Yan SF, Xue H, Zhang P, Sun JT, et al (2014) Cucurbitacin I induces protective autophagy in glioblastoma in vitro and in vivo. J Biol Chem 289: 10607–10619. 10.1074/jbc.M113.528760 24599950 PMC4036180

[35] Salhia B, Tran NL, Symons M, Winkles JA, Rutka JT, et al (2006) Molecular pathways triggering glioma cell invasion. Expert Rev Mol Diagn 6: 613–626. 16824034 10.1586/14737159.6.4.613

[36] Kwiatkowska A, Symons M (2013) Signaling determinants of glioma cell invasion. Adv Exp Med Biol 986: 121–141. 10.1007/978-94-007-4719-7_7 22879067

[37] Furnari FB, Fenton T, Bachoo RM, Mukasa A, Stommel JM, et al (2007) Malignant astrocytic glioma: genetics, biology, and paths to treatment. Genes Dev 21: 2683–2710. 17974913 10.1101/gad.1596707

[38] Kerbel RS (2008) Tumor angiogenesis. N Engl J Med 358: 2039–2049. 10.1056/NEJMra0706596 18463380 PMC4542009

[39] Bellou S, Pentheroudakis G, Murphy C, Fotsis T (2013) Anti-angiogenesis in cancer therapy: Hercules and hydra. Cancer Lett 338: 219–228. 10.1016/j.canlet.2013.05.015 23707856

[40] Carmeliet P (2005) VEGF as a key mediator of angiogenesis in cancer. Oncology 69 Suppl 3: 4–10. 16301830 10.1159/000088478

[41] Chaudhry IH, O'Donovan DG, Brenchley PE, Reid H, Roberts IS (2001) Vascular endothelial growth factor expression correlates with tumour grade and vascularity in gliomas. Histopathology 39: 409–415. 11683943 10.1046/j.1365-2559.2001.01230.x

[42] Dunn IF, Heese O, Black PM (2000) Growth factors in glioma angiogenesis: FGFs, PDGF, EGF, and TGFs. J Neurooncol 50: 121–137. 11245272 10.1023/a:1006436624862

[43] Bao S, Wu Q, Sathornsumetee S, Hao Y, Li Z, et al (2006) Stem cell-like glioma cells promote tumor angiogenesis through vascular endothelial growth factor. Cancer Res 66: 7843–7848. 16912155 10.1158/0008-5472.CAN-06-1010

[44] Machein MR, Plate KH (2000) VEGF in brain tumors. J Neurooncol 50: 109–120. 11245271 10.1023/a:1006416003964

[45] Ferrara N, Gerber HP (2001) The role of vascular endothelial growth factor in angiogenesis. Acta Haematol 106: 148–156. 11815711 10.1159/000046610

[46] Dvorak HF, Detmar M, Claffey KP, Nagy JA, van de Water L, et al (1995) Vascular permeability factor/vascular endothelial growth factor: an important mediator of angiogenesis in malignancy and inflammation. Int Arch Allergy Immunol 107: 233–235. 7542074 10.1159/000236988

[47] Dvorak HF, Nagy JA, Feng D, Brown LF, Dvorak AM (1999) Vascular permeability factor/vascular endothelial growth factor and the significance of microvascular hyperpermeability in angiogenesis. Curr Top Microbiol Immunol 237: 97–132. 9893348 10.1007/978-3-642-59953-8_6

[48] Zachary I, Gliki G (2001) Signaling transduction mechanisms mediating biological actions of the vascular endothelial growth factor family. Cardiovasc Res 49: 568–581. 11166270 10.1016/s0008-6363(00)00268-6

[49] Saraswati S, Agrawal SS (2013) Brucine, an indole alkaloid from Strychnos nux-vomica attenuates VEGF-induced angiogenesis via inhibiting VEGFR2 signaling pathway in vitro and in vivo. Cancer Lett 332: 83–93. 10.1016/j.canlet.2013.01.012 23348691

[50] Siegelin MD, Raskett CM, Gilbert CA, Ross AH, Altieri DC (2010) Sorafenib exerts anti-glioma activity in vitro and in vivo. Neurosci Lett 478: 165–170. 10.1016/j.neulet.2010.05.009 20470863 PMC3198851

[51] Ren Z, Schaefer TS (2002) ErbB-2 activates Stat3 alpha in a Src- and JAK2-dependent manner. J Biol Chem 277: 38486–38493. 11940572 10.1074/jbc.M112438200

