# Supplementary figures and images for: JSI-124 Suppresses Invasion and Angiogenesis of Glioblastoma Cells In Vitro
Source: PLoS One. 2015 Mar 19;10(3):e0118894. doi: 10.1371/journal.pone.0118894 (PMC4366361; doi:10.1371/journal.pone.0118894)

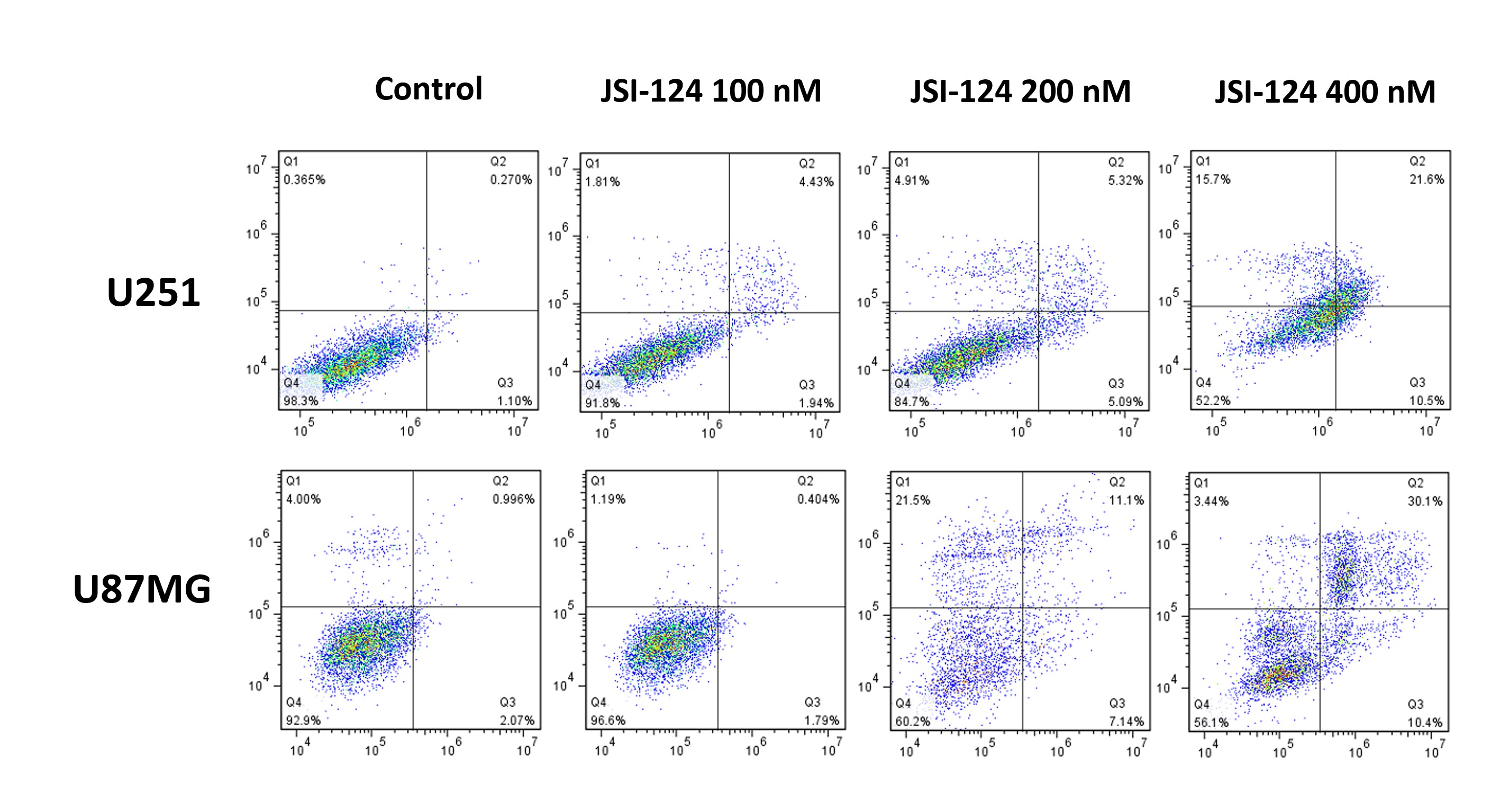

Supplement: S1 Fig — (TIF) [file pone.0118894.s001.tif]

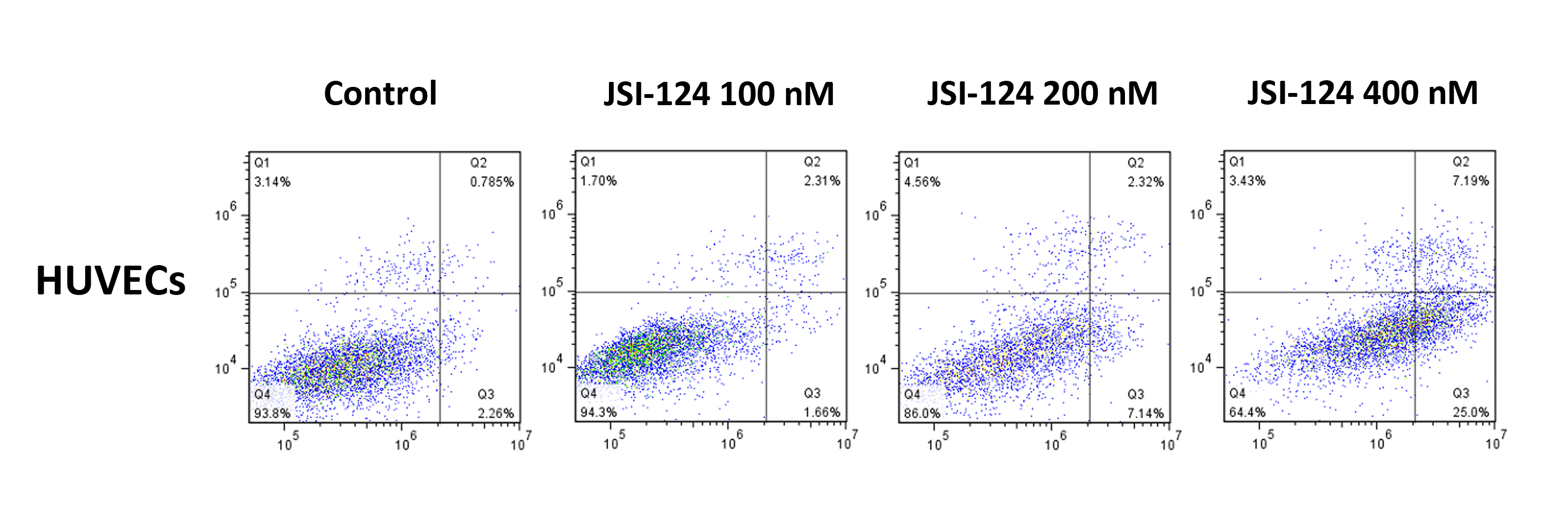

Supplement: S2 Fig — (TIF) [file pone.0118894.s002.tif]
